# Multi-Scale Modeling of Microstructure Evolution during Multi-Pass Hot-Rolling and Cooling Process

**DOI:** 10.3390/ma14112947

**Published:** 2021-05-29

**Authors:** Xian Lin, Xinyi Zou, Dong An, Bruce W. Krakauer, Mingfang Zhu

**Affiliations:** 1 Jiangsu Key Laboratory for Advanced Metallic Materials, School of Materials Science and Engineering, Southeast University, Nanjing 211189, China; xianlin@seu.edu.cn (X.L.); 220181923@seu.edu.cn (X.Z.); dong.an@seu.edu.cn (D.A.); 2 A. O. Smith Corporation, Milwaukee, WI 53224, USA; bkrakauer@aosmith.com

**Keywords:** hot-rolling, recrystallization, austenite to ferrite transformation, cellular automaton, finite element method (FEM)

## Abstract

In this work, a 6-pass hot-rolling process followed by air cooling is studied by means of a coupled multi-scale simulation approach. The finite element method (FEM) is utilized to obtain macroscale thermomechanical parameters including temperature and strain rate. The microstructure evolution during the recrystallization and austenite (γ) to ferrite (α) transformation is simulated by a mesoscale cellular automaton (CA) model. The solute drag effect is included in the CA model to take into account the influence of manganese on the γ/α interface migration. The driving force for α-phase nucleation and growth also involves the contribution of the deformation stored energy inherited from hot-rolling. The simulation renders a clear visualization of the evolving grain structure during a multi-pass hot-rolling process. The variations of the nonuniform, deformation-stored energy field and carbon concentration field are also reproduced. A detailed analysis demonstrates how the parameters, including strain rate, grain size, temperature, and inter-pass time, influence the different mechanisms of recrystallization. Grain refinement induced by recrystallization and the γ→α phase transformation is also quantified. The simulated final α-fraction and the average α-grain size agree reasonably well with the experimental microstructure.

## 1. Introduction

Grain refinement is a critical objective of the thermomechanical processing of Advanced High Strength Steels (AHSS). During hot-rolling, grain refinement is achieved primarily by microstructure evolution during austenite (γ) recrystallization and following the austenite to ferrite (γ→α) transformation [1]. Therefore, the understanding of microstructure evolution during recrystallization and the γ→α phase transformation is crucial for optimizing the rolling processes and enhancing the properties of products. Based on the time period that recrystallization takes place, there are three recrystallization mechanisms: (1) dynamic recrystallization (DRX)—nucleation and growth of recrystallized grains under deformation; (2) metadynamic recrystallization (MDRX)—growth of DRX grains during the inter-pass period; (3) static recrystallization (SRX)—nucleation and growth of recrystallized grain during the inter-pass period.

Extensive experiments have been carried out to study the microstructures and properties of AHSS under various rolling parameters, such as strain rate [2,3,4] and deformation temperature [5,6,7]. Those experimental studies provided important information concerning the relationship between process variables and grain structures after rolling. As a result of inherent experimental difficulties, these studies cannot fully elucidate the physical mechanisms contributing to grain refinement, because one needs to consider the temporal evolution of the multi-pass processes of recrystallization and the γ→α transformation, in order to analyze the contributions of recrystallization quantitatively, including DRX, MDRX, SRX, and the γ→α phase transformation.

With the development of technologies in computer science, numerical simulation methods have popularized and become an important tool for understanding the mechanisms of microstructure formation during material processes due to their capabilities to present the visual, temporal evolution of microstructures. Among different numerical models, the cellular automata (CA) approach, combining both computational efficiency and simplicity [8], has been commonly applied to investigate various phenomena such as recrystallization [9,10,11,12,13,14,15,16,17,18,19,20,21,22], phase transformation [23,24,25,26,27,28,29,30,31], and grain coarsening [32,33].

Simulation studies on microstructure evolution during recrystallization for a single-pass process have been performed by utilizing a CA model [9,10,11] and combining the CA method with the finite element method (FEM) [12,13] or the crystal plasticity finite element method (CPFEM) [14,15,16]. Those studies investigated the mesoscale grain structural evolution as well as the macro- or meso-scale mechanical response. However, there are few simulation studies that focused on multi-pass hot deformation processes using a coupled FEM and CA approach. Barkóczy et al. [17] first simulated the 4-pass rolling process using a CA method, considering SRX and grain coarsening. Yet, the simulation was based on unrealistic material parameters and an unspecific deformation process. The mechanism of dislocation density evolution was also not reproduced. Zheng et al. [18] and Chen et al. [19] input actual material and technology parameters to simulate a 7-pass hot-rolling process and 4-stage hot compression process. The effect of hot deformation was coupled through the determination of deformation-stored energy variation and a topology deformation technique for reproducing the plastically deformed grain structure. Good agreement of average grain size was obtained among the results from the CA simulation, the in-house software of ROLLAN, and a Gleeble simulator. Nevertheless, the simplified assumptions of temperature variation and mechanical response in those studies were insufficient due to the multi-scale nature of hot deformation. Svyetlichnyy et al. [20,21] simulated multi-pass shape rolling processes using a coupled FEM and frontal cellular automata (FCA) method. The results can be used to predict the grain structure and kinetics of recrystallization. However, the above-mentioned studies only focused on recrystallization during multi-pass hot deformation. Svyetlichnyy et al. [22] used a combined FEM and FCA model to simulate recrystallization during 3-pass shape rolling and a subsequent phase transformation during cooling. Nevertheless, an arbitrary α/γ grain boundary migration rate was used for phase transformation. The simulated microstructure was not validated by an experiment.

For the γ→α phase transformation in Fe-C-Mn alloys, the growth kinetics have been usually described by carbon diffusion and interface reaction based on the paraequilibrium condition, where only the interstitials are allowed to partition and reach the equality of chemical potentials between the α- and γ-phases, while the substitutional elements are not [34]. Several numerical simulation studies focused on the α-γ phase transformation during isothermal annealing [23,24], continuous cooling [25,26], continuous heating [27,28], and an entire anneal cycle [29,30,31] under the assumption of paraequilibrium. To account for the interaction of substitutional elements at the moving α/γ interface, Purdy et al. [35] proposed a solute drag model, where the Gibbs energy dissipation due to the trans-interface diffusion of the substitutional solute was introduced. An et al. [29] first coupled a solute drag model with a CA model to simulate the α-γ transformation in a dual-phase steel during different heat treatment processes. The simulation results agree well with those from phase field predictions, atom-probe tomography analyses, and SEM micrographs. However, a CA model incorporated with the solute drag effect for the simulation of the γ-α phase transformation has not been applied to the hot-rolling process.

As described above, at present, quantitative multi-scale simulation studies on both recrystallization and the γ→α phase transformation during a multi-pass hot-rolling process are still limited. In this article, a coupled macroscopic FEM and mesoscopic CA model is proposed to investigate the recrystallization and γ→α phase transformation during a 6-pass hot-rolling and continuous cooling process for an Fe-C-Mn steel. FEM is used to obtain the macroscopic thermomechanical data for CA simulations. The solute drag model is embedded in the CA approach to take into account the effect arising from the redistribution of manganese atoms at the α/γ interface. The evolution of the microstructure, dislocation density, and carbon concentration field during a specific multi-pass hot-rolling process is displayed. The effects of thermomechanical parameters on the recrystallization are discussed in detail.

## 2. Experiments and FEM Simulation

The steel with the composition Fe-0.07C-1.2Mn-0.5Si-0.052P-0.01S (wt.%) was hot-rolled using a large-scale laboratory hot-rolling mill with 1500 mm diameter work rolls. Before hot-rolling, the slabs were reheated to 1150 °C for 2 h. Then, the slab was deformed by a 6-pass hot-rolling process following by air cooling at a cooling rate of ~4 °C s^−1^ to room temperature. The rolling temperature at the start of each deformation pass, i.e., the initial rolling temperature, and the temperature at the end of the sixth pass are measured using a high-temperature handheld infrared thermometer (Table 1). The accuracy of the thermometer is about 1% of reading, and the max resolution is about 1 °C. The finishing rolling temperature after the sixth pass measured in the experiment is ~880 °C. The rolling process parameters are also presented in Table 1. The true strain in Table 1 is the plastic strain along the loading direction.

The FEM simulation of the continuous rolling process is conducted using the commercial software DEFORM-3D (v11.0.2, Scientific Forming Technologies Corporation, Columbus, United States). Process parameters and geometric models used for FEM simulation are the same as those in experiments (Table 1). The material file selected for the slab is modified based on tensile tests and thermomechanical analysis experiments. The outset of each deformation, also the end of each previous inter-pass, is determined as the time when the simulated temperature at the center of the slab’s upper surface is identical to the experimentally measured initial rolling temperature. The FEM simulation is finished when the simulated temperature at the center of slab upper surface equals the finishing rolling temperature of the sixth pass. Other thermophysical parameters are taken as follows. The convective heat transfer coefficient between the slab and air is 0.045 kW/m^2^⋅°C. The heat transfer coefficient between the slab and rolls is 9.5 kW/m^2^⋅°C. The coefficient of shear friction is 0.7.

## 3. Governing Equations and Numerical Methods of the CA Model

### 3.1. Model Description

A 2D mesoscopic CA model is proposed to simulate γ-recrystallization and the γ→α transformation during a 6-pass hot-rolling and subsequent air-cooling process. Recrystallization occurs during the 6-pass hot-rolling and continues until the temperature cools down to the transformation start temperature. The γ→α phase transformation takes place during the air-cooling process after 6-pass rolling.

The CA model of recrystallization simulation is divided into two parts. One part is for calculating the variation of dislocation density due to work hardening, recovery, and recrystallization. The other is for simulating the evolution of the plastically deformed microstructure under three recrystallization mechanisms, namely DRX, MDRX, and SRX, using the approach previously applied by Zheng et al. [18]. During the deformation period, the nucleation and growth of DRX grains occur. During the inter-pass interval, the growth of DRX grain, i.e., MDRX, as well as the nucleation and growth of SRX grains, takes place. A uniform topology mapping technique [36] is utilized to simulate the grain deformation structure. The strain rate and temperature fields are taken as homogeneous in the CA calculation domain, since the whole CA domain is within one element of FEM. The distribution of deformation energy within a grain is considered to be nonuniform with respect to the distance of a cell from a grain boundary.

The CA simulation for the γ→α phase transformation includes α-phase nucleation, α-grain growth and coarsening, and carbon diffusion, which is performed based on a quantitative CA model proposed by An et al. [29]. The multi-component steel used in the experiment is reduced to a ternary Fe-0.323C-1.231Mn (mol.%) alloy for simplicity. The assumption of paraequilibrium is adopted, where the partition of the substitutional element manganese at the γ/α interface is neglected. The solute drag effect of the element manganese is incorporated, which reproduces the decrease in grain boundary mobility due to manganese diffusion inside the α/γ interface. Moreover, the driving force for α-phase nucleation and growth also involves the contribution of the deformation-stored energy inherited from hot-rolling. Both the initial microstructure and deformation-stored energy field for phase transformation simulation are taken from those by the CA simulation at the end of the 6-pass hot-rolling. All thermodynamic data are obtained from Thermo-calc (TCFE9 database). The cooling rate is set as 4 °C/s as the rate measured in the experiment. The transformation temperature range is set from 832 °C to 650 °C based on thermal dilatometer measurements and kinetic factors.

In the CA model, space is discretized into a finite number of cells. Each cell is characterized by several state variables: (1) grain index, *I*; (2) dislocation density, *ρ_i_*_,*j*_; (3) average carbon concentration, *x*_C_; (4) α-phase volume fraction, *φ* (*φ* = 1 or 0 denotes the α- or γ-phase, respectively); (5) interface labels denote the recrystallized γ/unrecrystallized γ, γ/γ and α/α grain boundaries, and α/γ interfaces.

### 3.2. Austenite Recrystallization

#### 3.2.1. Dislocation Density Evolution

During hot deformation, most of the energy (~99%) is released immediately as heat. The residual energy remains stored in the form of dislocations. This deformation-stored energy (per volume), *E*_def_, can be calculated from the dislocation density, *ρ*, as follows [37]:

(1) $$E_{\mathrm{def}}=\beta \mu b^{2}\rho ,$$

where *β* is a constant of the order of 0.5; *μ* is the shear modulus of the γ-phase, 50 × 10^9^ Pa [23]; *b* is the magnitude of the Burgers vector, 2.48 × 10^−10^ m [23]. The relationship between the flow stress, *σ*, and the average dislocation density of the material, *ρ*_ave_, can be described as follows:

(2) $$\sigma =\alpha_{1}\mu b\sqrt{\rho_{\mathrm{ave}}},$$

where *α*_1_ is a constant depending on the dislocation/dislocation interaction, and can be taken as 0.5. The Kocks–Mecking (KM) model [38] is applied to evaluate the variation in the dislocation density in each grain with respect to deformation strain using the following equation:

(3) $$\frac{d\rho }{d\varepsilon }=k_{1}\sqrt{\rho }-k_{2}\rho ,$$

where *k*_1_ = 2*θ*_0_/(*α*_1_*μb*) is a constant representing work hardening; *k*_2_ = 2*θ*_0_/*σ*_s_ is the softening parameter representing dynamic recovery; *θ*_0_ is the initial work hardening rate; *σ*_s_ is the saturated stress, and it can be determined by Hatta’s model [39]:

(4) $$\dot{\varepsilon }=A_{0}{\left[\sin h\left(a\sigma_{s}\right)\right]}^{n^{\prime }}\exp\left(-\frac{Q_{A}}{RT}\right),$$

where $\dot{\varepsilon }$ is the strain rate; *A*_0_, *n*’, *a*, and *Q*_A_ are the material constants and can be obtained from flow stress-strain curve; *R* is the gas constant, 8.314 J mol^−1^ K^−1^; *T* is the absolute temperature. During the inter-pass intervals, static recovery occurs mainly via dislocation climbing. The decrease in the dislocation density due to static recovery can be expressed by [40]:

(5) $$\frac{d\rho }{dt}=d\;\cdot \;\left(\rho -\rho_{0}\right),$$

where *ρ*_0_ is the initial dislocation density and is set as the common dislocation density for annealed metals, 10^10^ m^−2^ [41]; *d* is a temperature-dependent coefficient representing the static recovery rate. It can be calculated by [40]

(6) $$d=d_{0}{\bar{D}}_{\gamma }^{md}\exp\left(-Q_{\mathrm{SRV}}/RT\right),$$

where ${\bar{D}}_{\gamma }$ is the average *γ*-grain diameter; *d*_0_, *md*, and *Q*_SRV_ are constants. In addition to recovery, recrystallization also accounts for dynamic and static softening. The dislocation density decreases in cell (*i*, *j*) caused by a recrystallized grain growing into a deformed grain can be expressed as

(7) $$\Delta \rho_{i,j}^{t}=\Delta f\left(\bar{\rho_{r}}-\bar{\rho_{\mathrm{nr}}}\right),$$

where $\bar{\rho_{nr}}$ is the average dislocation density of the deformed grain; $\bar{\rho_{r}}$ is the average dislocation density of the recrystallized grain; Δ*f* is the recrystallization fraction in cell (*i*, *j*).

A simplified analytical model [42] is adopted to reproduce the heterogeneous distribution of deformation energy within grains. The stored energy in cell (*i*, *j*) belongs to grain *S* and can be expressed as

(8) $$E_{S\left(i,j\right)}=f\left(L\right)H_{S,\;\mathrm{Max}}\;,\frac{1}{n}\;\sum_{i,j}E_{S\left(i,j\right)}=\bar{E_{S}},$$

where *H_S_*_,Max_ is the maximum value of the stored energy in grain *S*; *L* is the distance of the cell (*i*, *j*) from the grain boundary; *f*(*L*) is a factor decreasing from 1.0 to 0.2 in a length of 4.8 μm as *L* increases; *n* is the number of cells that belongs to grain *S*; $\bar{E_{S}}$ is the average deformation stored energy of grain *S*, which can be calculated from Equations (1) and (2).

#### 3.2.2. Nucleation of Austenite Recrystallization

The nucleation of recrystallization is assumed only to occur at austenite grain boundaries once the accumulation of dislocations reaches the critical dislocation density. Moreover, it is assumed to be a continuous nucleation event. During deformation, the nucleation rate per potential nucleation area for DRX as a function of both temperature *T* and strain rate $\dot{\varepsilon }$ is calculated by [43]

(9) $${\dot{n}}_{\mathrm{DRX}}=C{\dot{\varepsilon }}^{\eta }\exp\left(-\frac{Q_{N}}{RT}\right),$$

where *C* is the nucleation parameter, which can be estimated either by experiment or the inverse analysis method [44]. The value of *C* could be on the order of 10^12^–10^22^ [10,11,45]. In the present study, it is set to 7.2 × 10^15^ by comparing the simulation results with experimental data; *Q*_N_ is the activation energy for nucleation, 170 KJ mol^−^^1^ [23]; the exponent *η* is set to be 1 in the present simulation. The critical dislocation density *ρ*_c_ for DRX nucleation on grain boundaries is evaluated by [46]

(10) $$\rho_{c}={\left(\frac{20\gamma_{b}\dot{\varepsilon }}{3blM_{b}\tau^{2}}\right)}^{\frac{1}{3}},$$

where *γ*_b_ is high-angle grain boundary energy with a typical value of 0.56 J·m^−2^; *l* = 10.5 *μb*/*σ* is the dislocation mean free path [47]; *τ* = *μb*^2^/2 is the dislocation line energy; *M*_b_ is the high-angle grain boundary mobility, which can be expressed as [48]

(11) $$\;M_{b}=\frac{D_{0}b^{2}}{kT}\exp\left(-\frac{Q_{b}}{RT}\right),$$

where *D*_0_ is the boundary self-diffusion coefficient, 1.13 × 10^−^^6^ m^2^ s^−1^ [49]; *Q*_b_ is the activation energy for grain-boundary motion, 140 KJ mol^−^^1^ [18]; *k* is Boltzmann constant. During the inter-pass intervals, the nucleation rate of SRX per unit area in the deformed matrix is considered to be related to the distribution of deformation-stored energy *E*_def_ and temperature *T*. It is given by using a phenomenological model as follows [42]:

(12) $${\dot{n}}_{\mathrm{SRX}}=Z\left(E_{\mathrm{def}}-E_{c}\right)\exp\left(-\frac{Q_{N}}{RT}\right),$$

where *Z* is a nucleation parameter 1.389 × 10^10^; *E*_c_ the critical stored energy for initiating SRX, which can be determined from the critical deformation strain as follows [50]:

(13) $$E_{c}=\gamma_{b}\;\cdot \;10^{7}\left(\frac{\varepsilon_{c}}{2.2\varepsilon_{c}+1.1}\right),$$

where the critical strain *ɛ*_c_ generally ranges from 0.05 to 0.1 for different materials, and it can be taken as 0.1 for C-Mn steel [51].

#### 3.2.3. Grain Growth and Coarsening

The velocity of grain boundary movement, *V*, can be generally expressed as

(14) $$V=M_{b}P,$$

where *P* is the driving pressure for the specific process. For the recrystallization front moving into the deformed matrix, *P* is determined by the stored energy difference between the recrystallized grains and deformed matrix. For grain coarsening occurring at all grain boundaries, the driving force *P* is derived from curvature and is expressed as

(15) $$P=\gamma_{b}\kappa ,$$

where *κ* is the grain boundary curvature and calculated by [52]

(16) $$\kappa =\frac{A}{\Delta x}\;\frac{K_{\mathrm{ink}}-N_{S}}{N+1},$$

where ∆*x* is the CA cell size; *A* = 1.28 is a coefficient; *N* = 24 is the number of the first- and second-nearest neighbors; *N_S_* is the number of cells within the neighborhood belonging to the grain *S*; *K*_ink_ = 15 is the number of cells within the neighborhood belonging to grain *S* for a flat interface [52].

#### 3.2.4. Uniform Topology Deformation

For the present 2D model, the 2 × 2 uniform deformation matrix ***M*** is utilized to represent deformation. The transformation matrix ***M*** alters the original vector ***u*** to a new vector ***v***. It can be written as ***v*** = ***Mu***, or

(17) $$\left[\begin{array}{c} v_{x}\\ v_{y} \end{array}\right]=\left[\begin{array}{c} l_{x}\\ 0 \end{array}\begin{array}{c} 0\\ l_{y} \end{array}\right]\left[\begin{array}{c} u_{x}\\ u_{y} \end{array}\right],$$

where *u_i_* (*i* = *x*, *y*) and *v_i_* (*i* = *x*, *y*) are the components of the original vector ***u***, and the new vector ***v***; *l*_i_ (*i* = *x*, *y*) is equal to the ratios of the final to initial lengths of vectors along two principal axes. The volume is assumed to be consistent during deformation, which means that *l*_x_*l*_y_ = 1. Therefore, the true strain along the two principal axes of deformation can be written as

(18) $$\;\varepsilon_{i}=\ln l_{i}\left(i=x,y\right)$$

### 3.3. Austenite to Ferrite Transformation

#### 3.3.1. Ferrite Nucleation

During the cooling process after 6-pass hot-rolling, ferrite nucleates preferentially on the deformed γ-phase and γ-grain boundaries. The stored energy provides the additional driving force for ferrite nucleation. The classical nucleation theory is adopted to describe the nucleation rate of ferrite per unit in the potential nucleation area [53]:

(19) $$J=\frac{K_{1}D_{C}^{\gamma }}{\sqrt{kT}}\exp\left(-\frac{K_{2}}{kT\Delta G_{V}^{2}}\right),$$

where *K*_1_ is a constant related to the density of nucleation sites, and is taken as 2.48 × 10^1^^0^ J^1/2^ m^−^^4^ in the present work; *K*_2_ is a constant related to all the interfaces involved in nucleation, 2.5 × 10^−18^ J^3^ mol^−^^2^ [54], which relates to the shape of the nucleus and interfacial energy; $D_{C}^{\gamma }$ is the carbon diffusion coefficient in austenite; Δ*G*_V_ is the driving force for ferrite nucleation. It consists of both the chemical driving force, Δ*G*_V,che_, and the deformation stored energy, *E*_def_:

(20) $$\Delta G_{V}=\Delta G_{V,\mathrm{che}}+E_{\mathrm{def}}V_{m},$$

where *V*_m_ is molar volume of austenite, 7.18 × 10^−6^ m^3^ mol^−1^ [18]. Δ*G*_V,che_ is determined by the Gibbs chemical-free energy difference between the α- and γ-phases.

#### 3.3.2. Ferrite Growth and Coarsening

A mixed-mode growth model is adopted to describe the γ→α transformation, where the kinetics of ferrite growth are controlled by both carbon diffusion and α/γ interface mobility. The migration velocity of the α/γ interface *V*^α/γ^ is calculated by

(21) $$V^{\alpha /\gamma }=M^{\alpha /\gamma }P^{\alpha /\gamma },$$

where *P*^α/γ^ is the effective driving pressure; *M*^α/γ^ is the interfacial mobility of the moving α/γ interface, which can be described as [55]

(22) $$M^{\alpha /\gamma }=M_{0}^{\alpha /\gamma }\exp\left(-Q^{\alpha /\gamma }/RT\right),$$

where $M_{0}^{\alpha /\gamma }$ is the pre-exponential factor dependent on composition and processing history. It is adjustable and ranges from 1 × 10^−4^ to 0.5 mol m J^−1^ s^−1^ [29]. In the present study, $M_{0}^{\alpha /\gamma }$ is readjusted as 0.085 mol m J^−1^ s^−1^ based on the value estimated by Fazeli et al. [56] and by fitting the simulation results with the experimental micrograph; *Q*^α/γ^ is the activation energy for atom motion at the interface, 140 KJ mol^−1^; The effective driving pressure, *P*^α/γ^, involving the chemical driving pressure of the γ→α transformation, Δ*G*_che_, the solute drag pressure Δ*G*_dis_, and the deformation stored energy *E*_def_. *P*^α/γ^ is given by

(23) $$P^{\alpha /\gamma }=\Delta G_{\mathrm{che}}+E_{\mathrm{def}}V_{m}-\Delta G_{\mathrm{dis}},$$

where Δ*G*_dis_ is the dissipated Gibbs energy due to the solute drag effect, Section 3.3.3. Δ*G*_che_ can be calculated from

(24) $$\Delta G_{\mathrm{che}}=\chi \left(x_{C}^{\gamma ,\alpha /\gamma }-x_{C}^{\gamma ,e}\right),$$

where *χ* is a proportionality factor; $x_{C}^{\gamma ,e}$ is the equilibrium carbon concentration of the γ-phase, which can be obtained from thermodynamic calculation; $x_{C}^{\gamma ,\alpha /\gamma }$ is the actual carbon concentration of the γ-phase at the α/γ interface, which is obtained from the solute transport calculation, Section 3.3.4. To eliminate the artificial anisotropy that originated from the square CA cell, a geometrical factor *g*_new_ is introduced. It is related to the states of the neighboring cells and is defined by [29]

(25) $$g_{\mathrm{new}}=\;\min\left[1,\frac{1}{3}\left(\sum_{m=1}^{4}S_{m}^{I}+\frac{1}{\sqrt{2}}\sum_{m=1}^{4}S_{m}^{\mathrm{II}}\right)\right],S^{I},S^{\mathrm{II}}=\{\begin{array}{c} 0\;(\varphi <1)\\ 1\left(\varphi =1\right) \end{array},$$

where *S*^I^ and *S*^II^ indicate the states of the nearest neighbor cells and the second-nearest neighbor cells, respectively; *φ* is the α-phase volume fraction of the neighboring cells. Thus, the increment of the α-phase fraction during γ→α transformation is expressed as

(26) $$\Delta \varphi =-g_{\mathrm{new}}V^{\alpha /\gamma }\Delta t/\Delta x,$$

where Δ*t* is the time step. To reflect the higher kinetics of phase transformation along grain boundaries, the interfacial velocity along the γ/γ grain boundaries is assumed to be 2.5 times faster than that in other directions during cooling.

The α-grain coarsening is driven by curvature and can be found in Section 3.2.3.

#### 3.3.3. Solute Drag Model

The segregation of manganese at the α/γ interface would exert a solute drag pressure on the interface. A dissipated Gibbs energy, Δ*G*_dis_, is introduced to consider the velocity reduction of the grain boundary resulting from the solute drag effect. Δ*G*_dis_ can be calculated from the redistribution of manganese in the interfacial region, which is evaluated by [35]

(27) $$\Delta G_{\mathrm{dis}}=-\int_{-\Lambda }^{+\Lambda }\left(\;x_{\mathrm{Mn}}^{0}-x_{\mathrm{Mn}}\left(y\right)\right)\frac{dE\left(y\right)}{dy}dy\;,$$

where 2Λ is the physical interface thickness taken as 1 nm [57]; $x_{\mathrm{Mn}}^{0}$ is the manganese concentration in the bulk matrix; *x*_Mn_(*y*) is the manganese concentration profile across the interface; *E*(*y*) is the interaction potential of manganese with the interface; *y* is the distance from the interface. *E*(*y*) can be expressed as [35]

(28) $$E\left(y\right)=\{\begin{array}{c} \begin{array}{c} \mu_{\mathrm{Mn}}^{\alpha }\\ \mu_{\mathrm{Mn}}^{\alpha }+\Delta E-E_{0}+\frac{\left(\Delta E-E_{0}\right)}{\Lambda }y \end{array}\;\;\;\;\;\;\;\;\;\;\begin{array}{c} y<-\Lambda \\ -\Lambda \leq y<0 \end{array}\\ \begin{array}{c} \mu_{\mathrm{Mn}}^{\alpha }+\Delta E-E_{0}+\frac{\left(\Delta E+E_{0}\right)}{\Lambda }y\\ \mu_{Mn}^{\gamma } \end{array}\;\;\;\;\;\;\;\;\;\;\begin{array}{c} 0\leq y<+\Lambda \\ y\geq +\Lambda \end{array} \end{array}\;,$$

where 2Δ*E* is the potential difference in manganese between ferrite and austenite, which can be obtained from the thermodynamic calculation; *E*_0_ is the binding energy, i.e., the minimum in potential profile. It can be taken as 1.4RT − 24,000 J mol^−1^ [31]. *x*_Mn_(*y*) is given by [35]

(29) $$D_{\mathrm{Mn}}^{\mathrm{int}}\frac{\partial x_{\mathrm{Mn}}\left(y\right)}{\partial y}+\frac{D_{\mathrm{Mn}}^{\mathrm{int}}x_{\mathrm{Mn}}\left(y\right)}{RT}\frac{\partial E(y)}{\partial y}{+v}^{\alpha /\gamma }\left(x_{\mathrm{Mn}}{(y)-x}_{\mathrm{Mn}}^{0}\right)=0,$$

where $D_{\mathrm{Mn}}^{\mathrm{int}}$ = 1.42 × 10^−10^exp(−132000/*RT*) m^2^ s^−1^ is the diffusivity of solute manganese across the α/γ interface [56].

#### 3.3.4. Carbon Diffusion

Carbon partition and diffusion are governed by

(30) $$\partial x_{C}/\partial t=\nabla \cdot \left[D_{C}\left(\varphi \right)\cdot \;\nabla \left(x_{C}/p\left(\varphi \right)\right)\right],$$

where ${p(\varphi )=\varphi +k}_{e}\left(1-\varphi \right)$; $k_{e}{=x}_{C}^{\gamma ,e}{/x}_{C}^{\alpha ,e}$ is the equilibrium partitioning coefficient; $D_{C}\left(\varphi \right){=\varphi D}_{C}^{\alpha }{+k}_{e}{(1\;-\;\varphi )D}_{C}^{\gamma }$ is the carbon diffusion coefficient associated with the α-phase volume fraction, where $D_{C}^{\alpha }$, $D_{C}^{\gamma }$ are the temperature-dependent carbon diffusivities in the α- and γ-phases, respectively. They can be estimated by $D_{C}^{\alpha }=2.2\times 10^{-4}\exp\left(-122500/RT\right)$ m^2^ s^−^^1^ and $D_{C}^{\gamma }=1.5\times 10^{-5}\exp\left(-142100/RT\right)$ m^2^ s^−1^ [58]. Equation (30) is solved using the explicit finite difference scheme. The time step is determined by $\Delta t=\Delta x^{2}/\left(4.5D_{C}^{\alpha }\right)$. The zero-flux boundary condition is applied at the four walls of the calculation domain.

### 3.4. Coupling Scheme between CA and FEM Simulations

**Figure 1:**
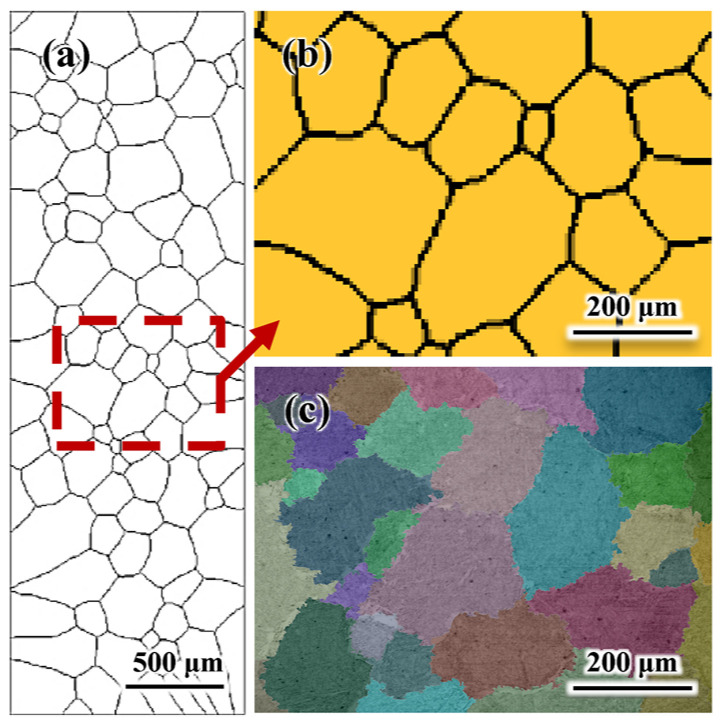
Initial grain structure before hot-rolling: (**a**) initial microstructure generated for the CA simulations; (**b**) enlarged microstructure in the region of the box in (**a**); (**c**) micrograph of the water-quenched sample cut from a slab after annealing at 1150 °C for 2 h before hot-rolling.

The initial grain structure for the CA simulation is generated through Voronoi tessellation according to the number and size of γ-grains measured from an experimental micrograph. The micrograph is obtained from a quenched sample after annealing at 1150 °C for 2 h. The initial microstructure for CA simulation, with an average grain size of ~174 μm, is generated based on the experimental microstructure (~179 μm), Figure 1. Similar grain sizes of the generated initial microstructure (~174 μm) and the experimental microstructure (~179 μm) are achieved, Figure 1. Macro-process parameters used in the CA simulation are obtained from the experiment and FEM simulations. The true strain for each pass in the CA simulation is identical to those in the hot-rolling experiment (Table 1). The temperature curve and the strain rate are obtained from the FEM simulation. The center element of the slab in the FEM calculation corresponds to the CA simulation domain. Moreover, the average effective strain rate of the center under deformation in FEM is taken for the CA simulation. Since the total reduction of the slab is extremely large (~94%), the calculation domain size reduces in each pass to improve the computational efficiency. Varying CA cell spacing is also required to accommodate the significantly changing average grain size in each pass. The domain size at the start of each deformation period and the CA cell spacing for each pass are listed in Table 2.

## 4. Results and Discussion

### 4.1. FEM Simulation of Hot-Rolling

First, FEM simulation using DEFORM-3D software is performed to obtain the average strain rate and the variations of the temperature field in the slab during the whole 6-pass hot-rolling process. Table 3 lists the simulated average effective strain rate, $\dot{\varepsilon }$, under each deformation period, which is in accordance with the variation of the true strain given in Table 1.

**Figure 2:**
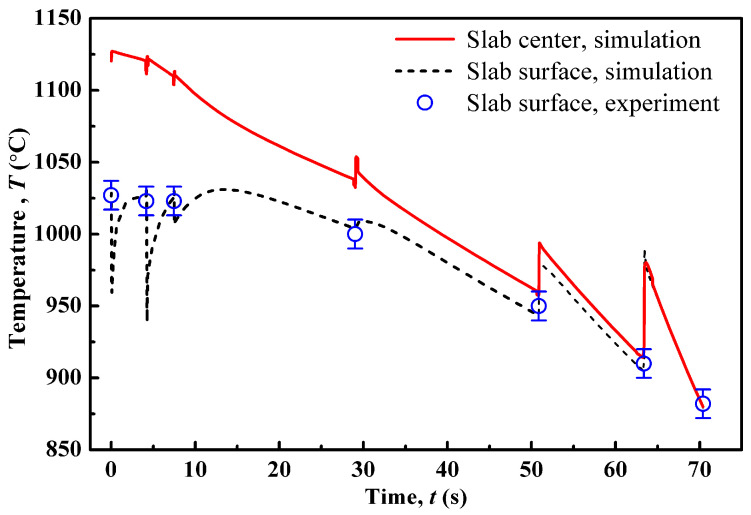
FEM-simulated temperatures of slab center (red line) and surface (black dash line) varying with time compared with the experimentally measured slab surface temperatures (blue circle).

Figure 2 displays the simulated temperature curves at the slab center and surface. In the curves, the sudden temperature changes imply the start of each pass. As shown, the simulated temperature at the surface is lower than that in the slab center by ~20–200 °C during the first to fourth pass, while the two temperature curves become nearly superposed in the last two passes, due to the fact that the rolled steel sheet becomes thin. During the hot-rolling process, the slab/sheet temperature is elevated by the heat generated under deformation. Simultaneously, at the slab surface, the temperature falls mostly by heat convection owing to the contact of slab/air and slab/rollers, while at the slab center, the temperature drops as a result of heat transfer from the center to surface. The surface temperatures measured in the experiment are also plotted with the simulated curve for comparison. However, the handheld pyrometer used in the experiments cannot determine the complete information on the slab temperature field. Therefore, it is necessary to perform FEM simulations to obtain the temperature field inside the slab.

### 4.2. CA Simulation of the Hot-Rolling Process

**Figure 3:**
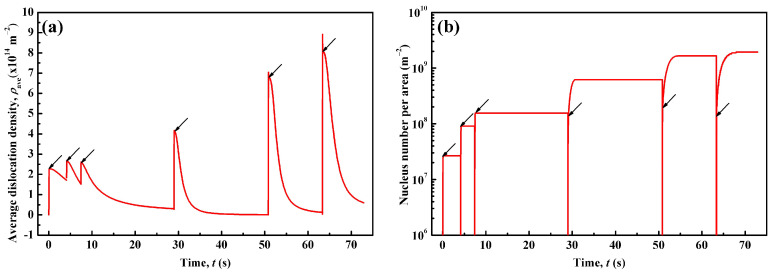
CA-simulated (**a**) average dislocation density and (**b**) recrystallized nucleus density varying with time. Arrows in the curves indicate the end of each pass.

The CA model described in Section 3.2 and Section 3.3 is applied to simulate the evolution of dislocation density and grain structure during the 6-pass hot-rolling process. The FEM-simulated temperature and strain rate are incorporated in the CA simulations. Figure 3 plots the simulated average dislocation density, *ρ*_ave_, and the recrystallized nucleus density varying with time. Arrows in the curves indicate the end of each pass. As shown in Figure 3a, the dislocation density increases rapidly under deformation and reaches a peak value in each pass. Then, it declines in the following inter-pass interval. For the whole process, the *ρ*_ave_ peak value shows an increasing trend with time except for the third pass. This corresponds to the strain rate with the pass number (Table 3), due to the fact that a higher strain rate enhances the dislocation density in the matrix. On the other hand, the *ρ*_ave_ valley value, representing the value of the remaining dislocation density in each pass, declines until the fourth interval. Especially at the end of the first and the second intervals, *ρ*_ave_ remains high (~1.5 × 10^14^ m^−2^) in the matrix and is inherited by the next pass. *ρ*_ave_ is almost reduced to zero at the end of the fourth interval, and then rises slightly afterwards. In the end of sixth pass before cooling, *ρ*_ave_ is in the order of 10^1^^3^ m^−2^, which is in the range of typical values for hot-rolled C-Mn steels [59].

In Figure 3b, the recrystallized nucleus density refers to the newly recrystallized nuclei in each pass, and thus it is zero at the start of each pass. As seen in each pass, the nucleus density increases rapidly. The steady value of the recrystallized nucleus density increases with the pass number. It is noted that there are almost no new nuclei appearing during the first to third inter-pass intervals, implying that the recrystallized nuclei are nearly produced by DRX during the former three passes. However, during the fourth to sixth inter-pass intervals, the nuclei number is increased, which is apparently generated by the SRX mechanism. The proportion of SRX to DRX nuclei also increases with the pass number. As shown in Figure 3a, the peak value of average dislocation density, *ρ*_ave_, gradually increases during the fourth to sixth pass. Obviously, higher *ρ*_ave_ in the fourth to sixth pass exceeds the critical value for SRX nucleation and leads to a larger recrystallization nucleation rate, Equations (12) and (13). Thus, it is understandable that SRX could happen and produce more nuclei in the latter three intervals. On the other hand, it is interesting to note that there is an increase in the DRX nucleus density from the first to third pass, although the variation of *ρ*_ave_ is nearly the same in those passes. It is considered that the initial grain size could account for this phenomenon [60,61,62]. Finer primary grains could provide more grain boundary area as potential nucleation sites, leading to a higher nucleus density in the matrix.

**Figure 4:**
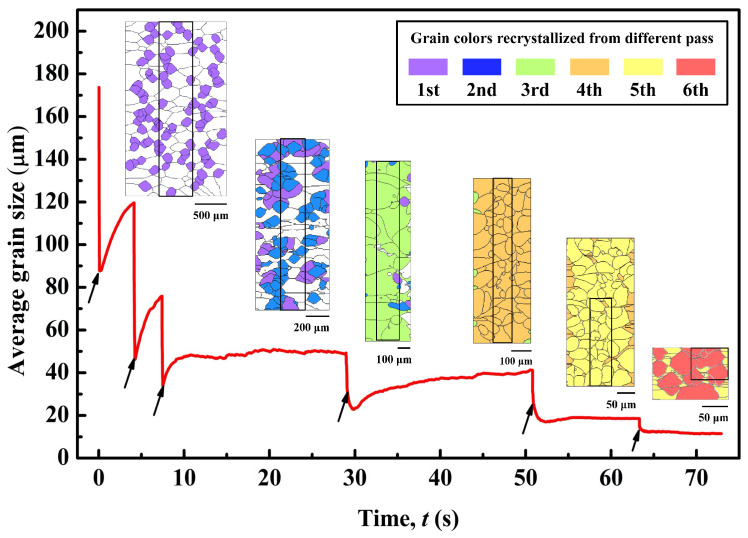
CA-simulated average grain size varying with time. Arrows indicate the end of each pass. Pictures from the left to right are the simulated grain structures at the end of each interval. The selected areas inside boxes shown in each picture are the input microstructures for the next pass. The sizes of each squared area are listed in Table 2. Recrystallized grains formed in different passes and intervals are shown in different colors; the initial matrix before the 1st pass is shown in white color.

Figure 4 presents the CA-simulated variation of average grain size and the grain structures at the end of each interval during the 6-pass hot-rolling process. The equivalent grain diameter is calculated using

(31) $$D=\frac{1}{N_{g}}\sum_{i=1}^{N_{g}}\sqrt{\frac{4A_{Si}}{\pi }},$$

where *N*_g_ is the number of grains in the calculation domain; *A_Si_* is the area of grain *S_i_*. It is seen in Figure 4 that for the first to third pass and interval, the average γ-grain size first reduces rapidly under deformation and then, as indicated by the arrows, rises immediately after finishing deformation. However, during the latter three intervals, the grain size is still reduced after deformation. As analyzed above, during the former three passes, the recrystallized nucleus is generated primarily by the DRX mechanism, while in the latter three passes and intervals, both DRX and SRX occur. Thus, in each pass, the dramatic reduction in average grain size results from the rapid nucleation of DRX. During the fourth to sixth intervals, the continuous decrease in average grain size after deformation results from SRX nucleation. The increase in grain size during the inter-pass period is due to the growth and coarsening of recrystallized grains.

Table 4 lists the average grain size of each type of grain at the end of each interval. The matrix grain refers to the grain structure inherited from the previous pass; DRX grain, formed by DRX and MDRX mechanisms, and SRX grain refer to the newly recrystallized grains in each pass. As shown, γ-grains are gradually refined from the initial ~174 μm to ~12 μm at the end of the sixth interval. At the end of each interval, the average matrix grain size (${\bar{D}}_{M}$), average DRX grain size (${\bar{D}}_{\mathrm{DRX}}$), and average SRX grain size (${\bar{D}}_{\mathrm{SRX}}$) are smaller than the average γ-grain size (${\bar{D}}_{\gamma }$) of the previous pass, which results in the refinement of the overall γ-grains. Each type of grain is also refined step by step except that ${\bar{D}}_{\mathrm{DRX}}$ increases at the end of the third interval. At the end of the first and second intervals, the limited inter-pass time and relatively low strain rate constrain the growth of DRX grains, i.e., MDRX mechanism, which leads to a smaller ${\bar{D}}_{\mathrm{DRX}}$ than ${\bar{D}}_{M}$. In the case of the third pass and interval, however, there is adequate inter-pass time for sufficient DRX grain structure development. Accordingly, the deformed matrix is greatly consumed. At the end of the third interval, ${\bar{D}}_{\mathrm{DRX}}$ is much larger than ${\bar{D}}_{M}$ and ${\bar{D}}_{\gamma }$, leading to a weakening of grain refinement and grain size uniformity. In the fourth to sixth pass, the strain rate is high enough (Table 3), and high stored energy accumulates in the matrix, which drives SRX grain growth. SRX grain growth would consume both DRX and matrix grains. At the end of the fourth to the sixth intervals, the average SRX grain size is largest. It is noted that, at the end of the sixth interval, no DRX grains remain, which means that DRX grain structure is totally replaced by SRX grains. The high strain rate in the sixth pass induces high stored energy remaining in the deformed matrix and DRX grains, and thus introduces a high difference in stored energy between SRX grains and other grain structures. The difference in stored energy provides a high driving force for SRX growing into matrix and DRX grains. Therefore, the SRX mechanism could take advantage of the high strain rate when competing with the MDRX mechanism.

**Figure 5:**
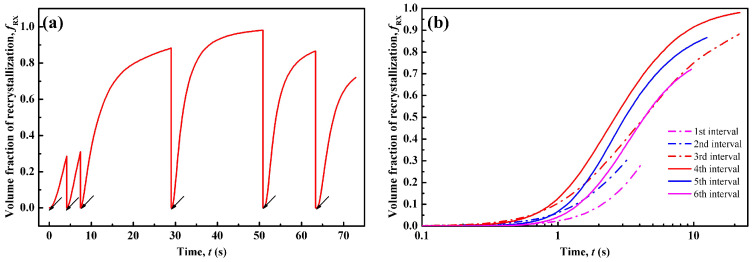
CA-simulated volume fraction of recrystallization varying with time: (**a**) an overview of the 6-pass hot-rolling process and (**b**) in each pass. Arrows in (**a**) indicate the end of each pass.

Figure 5 shows the recrystallization volume fraction, *f*_RX_, varying with time. In Figure 5a, *f*_RX_ refers to the volume percentage of the newly recrystallized grains during each pass, and thus it is zero at the start of each pass. During the first to fourth intervals, *f*_RX_ increases gradually and approaches a nearly saturated value of ~1.0 at the end of fourth interval. Then, *f*_RX_ decreases in the fifth and sixth intervals. As indicated by the arrows, the *f*_RX_ increment is not evident during the deformation period. The obvious *f*_RX_ increase appears during the inter-pass period. This is because the deformation time is extremely short (~0.1 s), and thus the growth of DRX nuclei is quite limited during deformation. Rapid development of the recrystallized structure takes place during the inter-pass intervals. Therefore, MDRX and SRX are the controlling mechanisms for the recrystallized grain structure formation. As discussed, regarding Figure 3b and Figure 4, there is no SRX occurring during the first to third interval. Therefore, MDRX provides the main contribution for recrystallization during the former three intervals. For the latter three intervals, MDRX and SRX dominate concurrently.

Figure 5b compares the recrystallization kinetics of each inter-pass interval. It is seen that the kinetics of recrystallization accelerate gradually from the first to fourth pass. The kinetics curve of the fourth interval becomes the highest. However, from the fifth to the sixth interval, the kinetics are reduced. Numerous experiments found that recrystallization kinetics are influenced by various factors, including strain rate [3,4], temperature [7], initial grain size [60,61,62], as well as inter-pass time. It is obvious that a higher strain rate enhances recrystallization by introducing more dislocations and thus provides a higher nucleation rate and driving force for the growth of recrystallized grains. The increasing temperature also accelerates the kinetics by providing higher boundary mobility. The influence of initial grain size relates to the nucleation of both DRX and SRX. As shown in Figure 3b, the nuclei density increases gradually from the first to third pass, which is considered to result from gradually finer initial grain size. This leads to the acceleration in kinetics of MDRX in the former three passes. For the fourth interval, in addition to the effects of finer grains and increased strain rate, the SRX mechanism also contributes to the higher kinetics than in the third interval by generating more nuclei. Owing to the decreased temperature, the recrystallization kinetics of the last two intervals slow down, even though the strain rate is increased, and the grains are refined. Therefore, the recrystallization fraction in the fourth interval is the highest under the comprehensive effect of initial grain size, strain rate, temperature, as well as inter-pass time.

**Figure 6:**
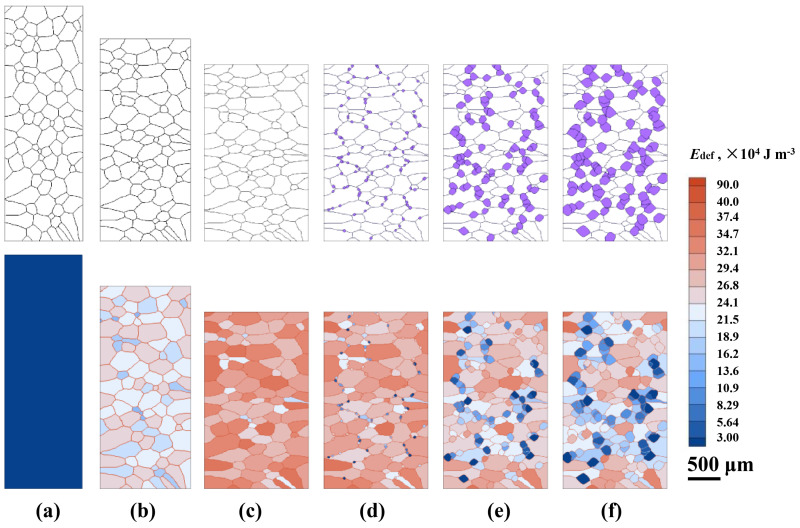
CA simulated evolution of the grain structure (upper row) and deformation-stored energy (lower row) during the 1st pass and interval: (**a**) *t* = 0.0 s; (**b**) *t* = 0.05 s; (**c**) *t* = 0.09 s, the end of deformation; (**d**) *t* = 1.0 s; (**e**) *t* = 3.0 s; (**f**) *t* = 4.1 s. In the upper row, the white and purple grains represent the initial and recrystallized grains generated in the 1st pass and interval, respectively. The horizontal and vertical directions are the rolling and compression directions, respectively.

The first and the fourth passes are taken as examples to explain the variations in grain structure and the deformation-stored energy (*E*_def_) field. Figure 6 presents the CA-simulated evolution of grain structure and the *E*_def_ field during the first pass and subsequent inter-pass period. As presented in Figure 6a, before hot-rolling, the initial grain structure is produced according to the experimental observation (see Figure 1). The initial *E*_def_ at grain boundaries is set to be somewhat higher than that within the grains, Equation (8). The deformed grain structures and nonuniform distribution of the *E*_def_ are achieved during deformation, Figure 6b,c. Figure 6c shows the grain structure and the *E*_def_ field at the end of deformation. It is seen that recrystallization does not occur evidently during the deformation period, reflecting the minor variation of *f*_RX_ in Figure 5a due to the limited deformation time. The *E*_def_ accumulates continuously under deformation, which corresponds to the increase in *ρ*_ave_ in the first pass (Figure 3a). During the inter-pass period, recrystallized grains appear at the γ/γ grain boundaries. As analyzed above, these recrystallized grains nucleate by the DRX mechanism. Then, as described by Equation (14), they grow into unrecrystallized grains by consuming the *E*_def_ during the inter-pass interval, Figure 6c–f. The *E*_def_ in some recrystallized grains is higher because they are generated prior to other recrystallized grains and thus accumulate more deformation-stored energy. It is also noted that the unrecrystallized grains surrounded by DRX grains have a relatively lower *E*_def_ than that far away from DRX grains, as the *E*_def_ in those grains is consumed by massive recrystallization, Equation (7).

**Figure 7:**
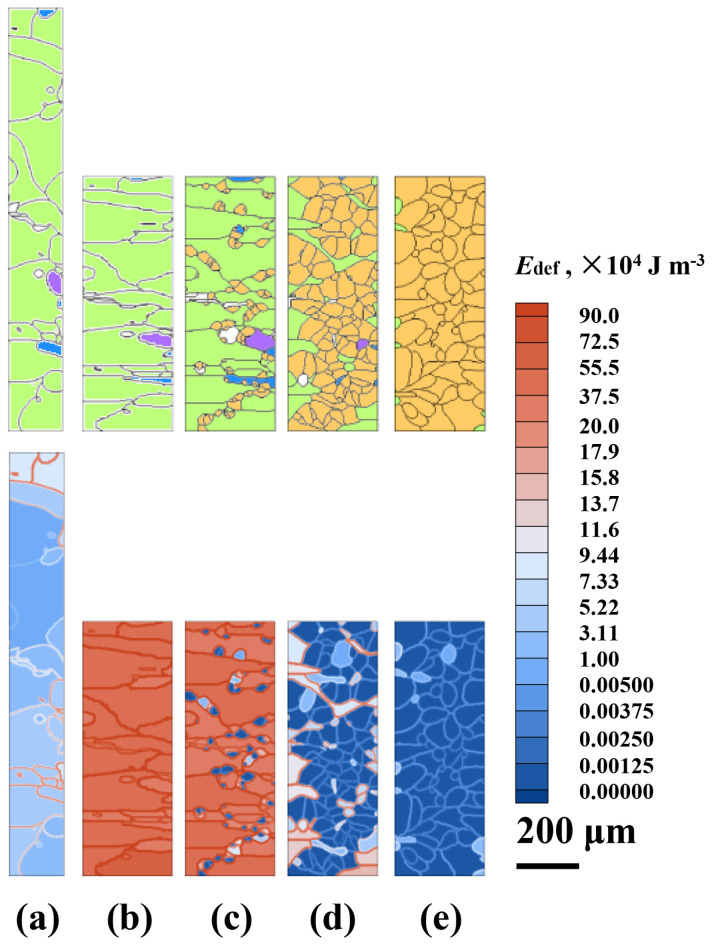
CA-simulated evolution of the grain structure (upper row) and deformation-stored energy (lower row) during the 4th pass and interval: (**a**) *t* = 0.0 s; (**b**) *t* = 0.1 s, the end of deformation; (**c**) *t* = 1.0 s; (**d**) *t* = 5.0 s; (**e**) *t* = 21.8 s. In the upper row, the purple, blue, green, yellow grains represent recrystallized grain generated in the 1st, 2nd, 3rd, 4th pass and interval, respectively. The horizontal and vertical directions are the rolling and compression directions, respectively.

Figure 7 presents the CA-simulated evolution of grain structure and the deformation-stored energy (*E*_def_) field during the fourth pass and interval. As shown, the nucleation from DRX can hardly be observed in Figure 7a,b, which is the same as that in the first pass. The *E*_def_ of the matrix at the end of the fourth pass deformation is much higher compared to that of the first pass. The remaining *E*_def_ after the fourth pass still exceeds the critical value for SRX nucleation, leading to the occurrence of SRX, Equation (12). Thus, during the inter-pass period, some new grains nucleate at both the unrecrystallized and recrystallized grain boundaries, Figure 7c–e. When the recrystallized grains contact each other, grain coarsening driven by curvature takes place, Equations (14)–(16). It is seen from Figure 7e that at the end of the fourth pass, the matrix has been almost thoroughly occupied by the new recrystallized grains, and the *E*_def_ in the domain has been exhausted to relatively low values. As discussed for Figure 5b, the nearly completely recrystallized grain structure after the fourth pass interval is due to an integrating effect of high strain rate (5.632 s^−1^), refined initial grain size (~40 μm), relatively long inter-pass time (~21 s), and moderate temperature (950–1050 °C).

### 4.3. CA Simulation of Cooling Process.

**Figure 8:**
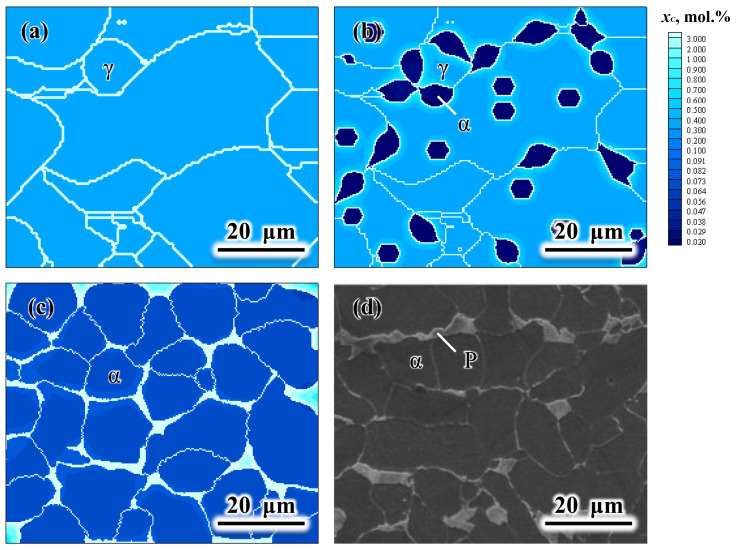
CA-simulated microstructures and carbon concentration fields during cooling from 832 °C to 650 °C at a cooling rate of 4 °C s^−1^: (**a**) *T* = 832 °C; (**b**) *T* = 750 °C; (**c**) *T* = 650 °C (*t* = 45.5 s, *f*_α_ = ~0.92, *D*_α_ = ~9.4 μm); (**d**) SEM micrograph (*f*_α_ = ~0.93, *D*_α_ = ~9.7 μm) of the cooled sample at room temperature. *f*_α_ is the α volume fraction, and *D*_α_ is the average diameter of α-grains.

Finally, the CA model coupled with the solute drag effect as described in Section 3.3 is adopted to simulate the microstructure evolution during cooling after the sixth pass. The grain structure at the end of sixth pass (inside the box of the last grain structure picture in Figure 4) is taken as the initial microstructure (Figure 8a) for this simulation. According to the Calphad calculation and experimental measurement, the γ→α transformation temperature range is set from 832 °C to 650 °C. Figure 8 displays the simulation results during cooling from 832 °C to 650 °C at a cooling rate of 4 °C s^−1^. The SEM micrograph from the as-rolled sample is given in Figure 8d for comparison. As shown in Figure 8b, new α-grains nucleate primarily at γ/γ grain boundaries; some α-grains also appear inside the γ-grains. During cooling, the α-grains grow with the increasing carbon concentrations in both α- and γ-phases. The regions adjacent to the α/γ interfaces are more enriched in carbon due to the rejection of carbon atoms from the newly formed α-grains. When the simulation of phase transformation finishes, carbon enriches in the remaining γ-phase (light blue) in Figure 8c, which corresponds to the pearlite phase in Figure 8d. The final α volume fraction of the simulated microstructure presented in Figure 8c is ~0.92, which is nearly identical to the experimentally measured value, ~0.93. The simulated average α-grain size, ~9.4 μm, also agrees well with the experimental data, ~9.7 μm.

## 5. Conclusions

A coupled macroscale finite element method (FEM) and mesoscale cellular automaton (CA) model is proposed for the simulation of microstructural evolution during a 6-pass hot-rolling process. The FEM is adopted to calculate the deformation and temperature field. The CA approach is used to simulate the dislocation density and microstructure quantitatively, including recrystallization during hot-rolling, and the γ→α phase transformation during cooling.

The calculation of the dislocation density field takes into account the effects of work hardening, dynamic recovery, static recovery, and recrystallization. For the simulation of the γ→α phase transformation, the interaction between manganese atoms and α/γ interfaces is considered by incorporating a solute drag effect with the CA model; the contribution of deformation-stored energy to the driving forces is also involved.

The simulation results quantitively display the comprehensive effect of strain rate, grain size, temperature, and inter-pass time on the different mechanisms of recrystallization, including dynamic recrystallization (DRX), metadynamic recrystallization (MDRX), and static recrystallization (SRX). DRX is found to be mainly constrained to the nucleation of recrystallized grains due to the limited deformation time (~0.1 s) in the present rolling process. Under the relatively low strain rate of the first to third passes, MDRX dominates the grain structure evolution. For the high strain rates of the fourth to sixth passes, MDRX and SRX occur concurrently. High strain rate is found to be more beneficial to SRX than MDRX. A high temperature and fine initial grain size will enhance the kinetics of recrystallization. A sufficient inter-pass time is also necessary for MDRX and SRX to refine grain structure. However, too long of an inter-pass duration may lead to recrystallized grains coarsening.

The simulated average γ-grain size is reduced from ~174 μm to ~12 μm during the 6-pass hot rolling process. After the γ→α phase transformation during subsequent cooling, the simulated final α volume fraction (0.92) and average α-grain size (~9.4 μm) agree reasonably with the experimental data (~0.93, ~9.7 μm). The simulation in the present work allows for quantification of the contributions of DRX, MDRX, SRX, and phase transformation on grain refinement, and thus provides insight into the mechanisms of microstructural evolution during a multi-pass hot-rolling process.

## Figures and Tables

**Table 1:** Strain and initial rolling temperature used for laboratory hot-rolling.

	Pass 1	Pass 2	Pass 3	Pass 4	Pass 5	Pass 6
Strain, *ε*	0.288	0.405	0.288	0.511	0.693	0.889
Initial rolling Temperature (°C)	1027 ± 10	1023 ± 10	1023 ± 10	1000 ± 10	950 ± 10	910 ± 10

**Table 2:** CA simulation settings.

	Pass 1	Pass 2	Pass 3	Pass 4	Pass 5	Pass 6	Cooling
Initial simulation domain size (CA cells)	250 × 750	111 × 563	110 × 752	73 × 565	80 × 680	100 × 400	240 × 200
CA space step, Δ*x* (μm)	4.8	4.8	2.4	2.4	1.2	0.6	0.3

**Table 3:** Average effective strain rate obtained from the FEM simulation.

	Pass 1	Pass 2	Pass 3	Pass 4	Pass 5	Pass 6
Strain rate, $\dot{\varepsilon }$ (s^−1^)	3.724	4.770	3.964	5.632	10.000	12.024

**Table 4:** Average grain size at the end of each interval.

Pass Number	Initial	1	2	3	4	5	6
Average grain size, ${\bar{D}}_{\gamma }$ (μm)	174	120	76	49	41	19	12
Average matrix grain size, ${\bar{D}}_{M}$ (μm)	174	147	87	27	20	12	8
Average DRX grain size, ${\bar{D}}_{\mathrm{DRX}}$ (μm)	--	93	63	72	33	15	--
Average SRX grain size, ${\bar{D}}_{\mathrm{SRX}}$ (μm)	--	--	--	--	45	23	19

## Data Availability

The data presented in this study are available in this article.

## Nomenclature

*γ*_b_	high-angle grain boundary energy: J m^−2^
*ε*	strain
*ɛ*_c_	critical strain for SRX nucleation
$\dot{\varepsilon }$	strain rate, s^−1^
*θ*_0_	initial work hardening rate, MPa
*κ*	the grain boundary curvature, m^−1^
Λ	half physical interface thickness, nm
*μ*	shear modulus of the γ-phase, Pa
*ρ*	dislocation density, m^−2^
*ρ*_0_	initial dislocation density, m^−2^
*ρ*_c_	critical dislocation density for DRX, m^−^^2^
*ρ*_ave_	average dislocation density of the material, m^−^^2^
$\bar{\rho_{nr}}$	average dislocation density of the deformed grain, m^−2^
$\bar{\rho_{r}}$	average dislocation density of the recrystallized grain, m^−2^
*σ*	flow stress, Pa
*σ*_s_	saturated stress, Pa
*τ*	dislocation line energy, J·m^−1^
*φ*	α-phase volume fraction
*χ*	proportionality factor, J mol^−^^1^ mol.%^−1^
*α*_1,_*β*, *η*, *a*, *n*’, *A*_0_, *Q*_A_	constants for calculating dislocation density evolution
*A*	coefficient for calculating curvature
*A_Si_*	area of grain *S_i_*
*b*	the magnitude of the Burgers vector, m
*C*	DRX nucleation parameter
*d*	coefficient representing static recovery rate
*d*_0_, *md Q*_SRV_	constants for calculating static recovery rate
${\bar{D}}_{\gamma },{\bar{D}}_{M},\;{\bar{D}}_{\mathrm{DRX}},{\bar{D}}_{\mathrm{SRX}},\;{\bar{D}}_{\alpha }$	average diameters for γ-, matrix, DRX, SRX, α-grains, μm
$D_{C}^{\alpha },\;D_{C}^{\gamma }$	carbon diffusion coefficients in the α- and γ-phases, m^2^ s^−1^
*D*_0_	boundary self-diffusion coefficient, m^2^ s^−^^1^
$D_{\mathrm{Mn}}^{\mathrm{int}}$	manganese diffusion coefficient across the α/γ interface, m^2^ s^−^^1^
*E*_def_	deformation stored energy, J m^−3^
*E*_c_	critical deformation stored energy for SRX nucleation, J m^−3^
$\bar{E_{S}}$	average deformation stored energy of grain *S*, J m^−3^
*E*_0_	binding energy of manganese, J mol^−1^
Δ*E*	half potential difference of manganese between α- and γ-phases, J mol^−^^1^
*f*_RX_, *f*_α_	volume fractions of recrystallized grains and α grains
Δ*G*_V_	driving force of the ferrite nucleation, J mol^−1^
Δ*G*_V,che_	Gibbs chemical free energy difference between the α- and γ-phases, J mol^−^^1^
Δ*G*_che_	chemical driving force of the γ→α transformation, J mol^−1^
Δ*G*_dis_	solute drag pressure, J mol^−1^
*g*_new_	geometrical factor
*H_S_*_,Max_	maximum value of the stored energy in grain *S*, J m^−^^3^
*I*	grain index
*J*	nucleation rate of ferrite, m^−^^2^ s^−^^1^
*k*	Boltzmann constant, J K^−1^
*k*_e_	equilibrium partitioning coefficient
*k*_1__,_ *k*_2_	constants representing work hardening and dynamic recovery
*K*_1_ *K*_2_	constants for calculating the nucleation rate of ferrite
*K*_ink_	number of cells within the neighborhood belonging to grain *S* for a flat interface
*l*	dislocation mean free path, m
*l*_i_	ratio of the final to initial length of vector along corresponding axis
*L*	distance of the cell (*i*, *j*) from the grain boundary, μm
*f*(*L*)	factor for deformation stored energy distribution
***M***	deformation matrix for topology mapping
*M*_b_	high-angle grain boundary mobility, m^4^ J^−1^ s^−1^
*M* ^α/γ^	interfacial mobility of the moving α/γ interface, mol m J^−1^ s^−1^
$M_{0}^{\alpha /\gamma }$	pre-exponential factor, mol m J^−1^ s^−^^1^
${\dot{n}}_{\mathrm{DRX}},\;{\dot{n}}_{\mathrm{SRX}}$	nucleation rates for DRX and SRX, m^−2^ s^−1^
*n*	number of cells that belongs to grain *S*
*N*	number of the first and second nearest neighbors
*N_S_*	number of cells within the neighborhood belonging to grain *S*
*N*_g_	number of grains in the calculation domain
*P*	driving pressure, J m^−^^3^
*P* ^α/γ^	effective driving pressure for ferrite growth, J mol^−1^
*Q*_N_	activation energy for nucleation, KJ mol^−1^
*Q*_b_	activation energy for grain-boundary motion, KJ mol^−^^1^
*Q* ^α/γ^	activation energy for atom motion at the interface, KJ mol^−1^
*R*	universal gas constant, J mol^−1^ K^−1^
*S*^I^, *S*^II^	states of the nearest and the second-nearest neighbor cells
*T*	absolute temperature, K
***u***_,_ ***v***	original and the new vectors of the CA cell space
*V*	velocity of grain boundary movement, m s^−1^
*V* ^α/γ^	migration velocity of the α/γ interface, m s^−1^
*V*_m_	molar volume of austenite, m^3^ mol^−1^
$x_{C}^{\alpha ,e}$	equilibrium carbon concentration of the α-phase, mol.%
$x_{C}^{\gamma ,e}$	equilibrium carbon concentration of the γ-phase, mol.%
$x_{C}^{\gamma ,\alpha /\gamma }$	actual carbon concentration of the γ-phase at the α/γ interface, mol.%
$x_{\mathrm{Mn}}^{0}$	manganese concentration in the bulk matrix, mol.%
*y*	distance from the interface, μm
*x*_Mn_(*y*)	the manganese concentration profile
*E*(*y*)	the interaction potential of manganese
*Z*	nucleation parameter for SRX, J^−1^ s^−1^
